# The Effect of an Online Sugar Fact Intervention: Change of Mothers with Young Children

**DOI:** 10.3390/nu12061859

**Published:** 2020-06-22

**Authors:** Yi-Chun Chen, Ya-Li Huang, Yi-Wen Chien, Mei Chun Chen

**Affiliations:** 1 School of Nutrition and Health Sciences, Taipei Medical University, Xinyi District, Taipei City 110, Taiwan; ychien@tmu.edu.tw (Y.-W.C.); gshejenny@gmail.com (M.C.C.); 2 Research Center of Health Equity, College of Public Health, Taipei Medical University, Xinyi District, Taipei City 110, Taiwan; ylhuang@tmu.edu.tw; 3 Department of Public Health, School of Medicine, College of Medicine, Taipei Medical University, Xinyi District, Taipei City 110, Taiwan; 4 School of Public Health, College of Public Health, Taipei Medical University, Xinyi District, Taipei City 110, Taiwan; 5 Graduate Institute of Metabolism and Obesity Sciences, College of Nutrition, Taipei Medical University, Xinyi District, Taipei City 110, Taiwan

**Keywords:** online nutrition intervention, theory of planned behavior, nutrition labels, sugar, consumer behavior, consumer attitude, consumer perception

## Abstract

Research indicates that high sugar intake in early childhood may increase risks of tooth decay, obesity and chronic disease later in life. In this sugar fact study, we explored whether an online intervention which focused on comprehensive and useful information about nutrition labels impacted mother’s choice of low sugar food. The intervention was developed on the basis of the theory of planned behavior. In total, 122 mothers were recruited. Mothers were divided into an online-only group and a plus group. Knowledge of sugar and nutrition labels, behavioral attitudes, perceived behavioral control, behavioral intentions and behavior towards purchasing low-sugar products with nutrition labels were collected. After the intervention, both groups exhibited significantly enhanced sugar and nutrition label knowledge, perceived behavioral control, behavioral intentions and behavior. Compared to the online-only group, knowledge, perceived behavioral control and behavior of the plus group significantly improved. After the intervention, about 40% of the plus group and 80% of the online-only group still did not know the World Health Organization (WHO) sugar recommendations. Understanding sugar recommendations and using nutrition labels are crucial to help people control calorie and sugar intake. Further research with a larger sample is warranted to evaluate the effects of the intervention on long-term changes in shopping behavior. More efficient and convenient nutrition education is required to increase public awareness of sugar recommendations and help people control calorie and sugar intake.

## 1. Background

Being overweight and obese increases the risks of many health problems, including diabetes, heart disease and certain cancers [1,2,3]. An examination of the 2015 data of the World Obesity Federation reveals that overweight rates of children in Taiwan were the highest in Asia [4]. A long-term follow-up study in Taiwan found that approximately 90% of young children consume sugary drinks and snacks once per day, and one-third of 5 year-old children more than 10% of their caloric intake from refined sugar [5]. Studies have confirmed that sugar promotes a high energy balance and children who consume more sugar have higher obesity rates than those who consume less sugar [6,7,8]. Thus, the World Health Organization (WHO) strongly recommends that it is good to reduce the sugar intake to <10% of total energy intake for both adults and children [9].

Products with low- or no-sugar-related claims, such as “sugar free”, “no added sugar” and “reduced sugar”, may be particularly appealing to parents who want to manage their child’s sugar intake [10,11]. However, a Canadian study found that half of 3048 prepackaged foods with sugar-related claims contained excessive sugar, and a greater proportion contained sweeteners than did products without such claims [12]. A survey in Taiwan also found that more than 90% of popular snacks and drinks with no-added-sugar claims consumed by children were high in sugar [13]. A study in Australia and New Zealand found that 28% of consumers misunderstood the meaning of the claim of “no added sugar”, believing that products with such a claim contained no sugar [14]. In addition, 95.7% of mothers know that excessive sugar intake increases future health risks in children, but only 21.9% know the WHO sugar recommendations [15].

Nutrition labeling is an important tool to help people choose healthy foods. A previous study has discovered that although most consumers trust nutrition labels, they perceive that the information on them is difficult to understand and confusing, including information on recommended daily allowances, percent daily values and servings [16]. Because of limited time, consumers normally only read one or two of the facts on nutrition labels, such as calories and fats [17]. The Health Information National Trends Survey in the US suggested that although most people have difficulty interpreting nutrition labels, they cannot effectively utilize such facts to make informed dietary choices they possess insufficient reading comprehension and calculation skills [18]. Education can help the public improve those skills required to understand nutrition labels, thereby allowing consumers to effectively purchase suitable foods according to nutrition labels [19]. A study in the US applied a multimedia intervention to participants in an experimental group and significantly improved their comprehension of nutrition labels [20]. Another study in the US demonstrated that online courses helped the public improve skills required to effectively use nutrition labels to buy healthy foods [21]. In brief, effective education can improve people’s comprehension of nutrition labels, thereby enabling the public to select healthy foods.

Despite the desire of parents to maintain their children’s optimal health, they may not provide healthy foods to their children if they lack nutrition knowledge. A survey of Taiwanese mothers with young children demonstrated that the less sugar-related knowledge they had, the more positive their attitude was toward no-added-sugar infant cereal and the higher their purchase intention was for these infant cereals [15]. Moreover, some parents who lack this knowledge consider providing healthy diets to their children a challenge [22]. Under such circumstances, desirable nutrition education can improve parents’ beliefs and behaviors concerning their children’s diets, allowing them to provide a healthy diet for their children. The theory of planned behavior (TPB) has been widely adopted in studies that explore or predict various health behaviors as well as those that explore health promotion. This theory posits that behavioral intentions are a pivotal antecedent that affects actual behaviors; furthermore, attitudes toward a behavior, subjective norms and perceived behavioral control are possible controlling factors that affect behavioral intentions [23]. Increasing one’s relevant knowledge and developing relevant skills help to change attitudes toward a specific behavior and in turn enhance individuals’ intentions to perform such a behavior, thereby encouraging individuals to improve that behavior. A study of Australian mothers with preschool-aged children demonstrated that perceived behavioral control and intention were positively associated with mothers’ healthy feeding behavior perceived behavioral control was the only variable positively associated with the mothers’ perceptions of their children’s fruit and vegetable consumption [22]. A study in South Australia indicated that a parent-focused nutrition intervention can affect maternal feeding practices, which reduced growth-related indicators of future obesity risk in young children [24]. Similarly, an Australian study regarding breakfast consumption indicated that people’s attitudes and perceived behavioral control significantly affected their intention to eat breakfast; such an intention affected an individual’s breakfast consumption [25]. A podcast-based research study in the US determined that listening to a podcast about omega-3 fatty acids in the grocery store enhanced customers’ attitudes and perceived behavioral control toward purchasing foods rich in omega-3 fatty acids [26]. According to the aforementioned studies, it was assumed that nutrition educational interventions based on the TPB can promote the public’s health behaviors.

In Taiwan, nutrition labeling regulations regarding sugar content apply to the products manufactured after July 2015 [27] and sugar recommendation was available on 2018 [28]. Encouraging people to use nutrition labels is crucial. Traditionally, interventions targeting parents with young children which required face-to-face classes had low attendance and high dropout rates [29,30]. Online interventions are an efficient and cost-effective way to provide nutrition education. Previous studies confirmed that online nutrition educational programs should be considered to expand outreach and decrease barriers to attending traditional face-to-face classes [31,32]. According a 2017 Taiwanese Internet usage survey, there was an 80% Internet usage rate across the nation [33]. Therefore, online education is feasible in Taiwan. In the present study, the TPB was used to develop an online intervention program to enhance mothers’ use of nutrition labels to buy low-sugar foods for their children.

## 2. Methods

### 2.1. Design and Participants

This Sugar Fact intervention was a quasi-experimental trial, which was conducted from December 2017 to August 2018 in Taiwan. Online videos and a small-group discussion were used to encourage mothers to use nutrition labeling to buy low-sugar foods for their children. The intervention was designed for the senior high school level because 95% of Taiwanese women aged 25–44 years have at least a senior high school education [34]. Mothers who live in Taiwan, communicate in Chinese, had a child aged 1–6 years, and were their child’s primary caregiver and the family’s food purchaser were eligible for the study. Various parenting social networks (e.g.; BabyHome, a mothers’ groups on Facebook and BabyMother on a bulletin board system) were approached and those who agreed to distribute or advertise the study posted a link to the online recruitment questionnaire on their network. Participants were classified into an online-only group or a plus group according to their intentions. An official Line account (a social networking app popular in Taiwan) was used to contact and follow-up participants. A notification message by Line was sent once per week to remind participants to continue the intervention. The intervention for the online-only group consisted of watching two online videos. The intervention of the plus group included watching two online video and participating in one small-group discussion.

**Figure 1:**
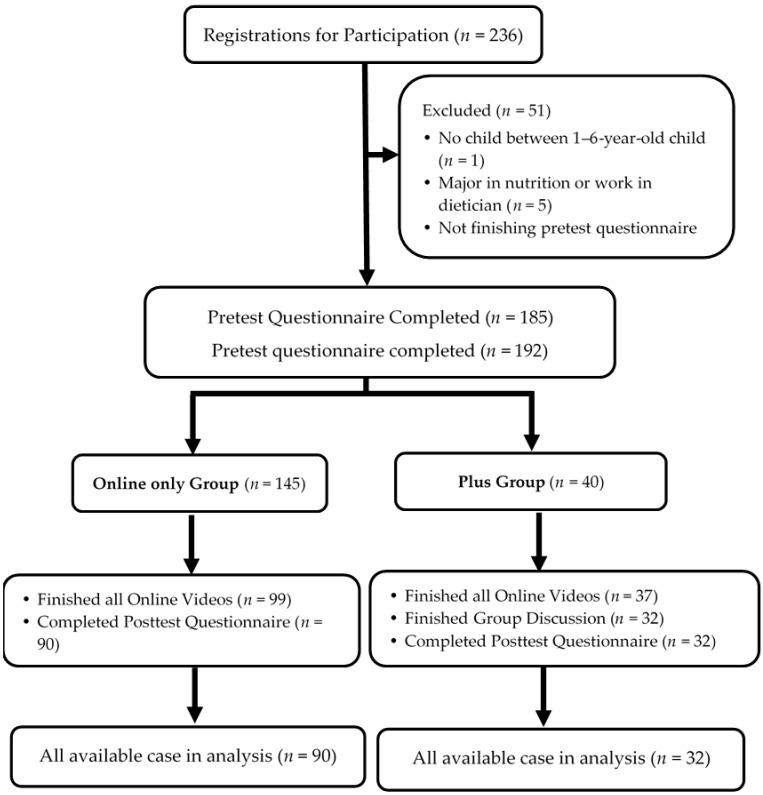
Recruitment of participants.

In total, 236 mothers were recruited, and 185 mothers were enrolled in the intervention. Finally, 90 mothers in the online-only group and 32 mothers in the plus group completed the intervention and posttest questionnaire (Figure 1). The completion rate was 62.1% for the online-only group and 80% for the plus group. Participants who completed the intervention received a commercial voucher as an incentive (NT$100 for the online-only group and NT $300 for the plus group; about US $3 and $9, respectively).

### 2.2. Ethical Considerations

The study was approved by the Taipei Medical University—Joint Institutional Review Board (N201711059) and written informed consent was obtained from all mothers. 

### 2.3. Developed Educational Intervention

**Figure 2:**
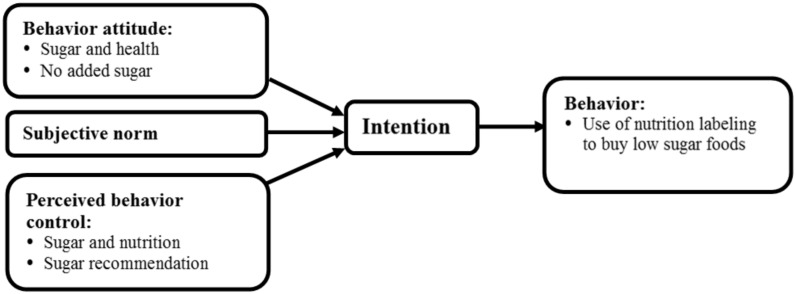
Intervention concept.

The educational intervention was developed based on results of a previous study [15]. Considering the difficulty of attending classes by mothers with young children [35], online video courses were used. The purpose of the online video session for mothers was to increase their positive attitudes and perceived behavioral control of using nutrition labels to buy low-sugar foods for their child (Figure 2). The purpose of the first video was to increase the mother’s perceptions of “sugar and health” and “sugar and nutrition”. The purpose of the second video was to improve the mothers’ understanding of “sugar recommendations”, “sugar-related claims” and “nutrition labeling on food packages”. All of the contents of the videos were reviewed by three public health nutrition professionals. Five mothers eligible for recruitment were included in a pilot intervention. They were asked to watch the first version of the two videos and give comments. The two videos were uploaded on YouTube after being revised.

For the online-only group, the nutrition intervention included two 15 min online video sessions. For the plus group, the nutrition intervention included two 15 min online video sessions and one small-group discussion led by the researchers (for 2–3 h). Additional educational materials included a booklet for the online videos and a pamphlet for the small-group discussion. The aims of the small-group discussion were to help mothers clarify the content of the online videos and improve their perceived behavioral control structure. The small-group discussion session focused on barriers that mothers encounter when selecting low-sugar foods and controlling their children’s sugar intake. From January to May 2018, the small-group discussion sessions were held 10 times. There were three to five mothers in each small-group discussion. In total, 32 mothers participated in small-group discussions, and all group discussions were recorded. Several topics on a list were discussed to gain an in-depth understanding of participants’ reactions to the online video courses and barriers when the mothers practiced their skills of choosing low-sugar foods for their children. The mothers were also allowed to practice using nutrition labels to choose low-sugar foods.

### 2.4. Questionnaire

A theory-based questionnaire containing the mother’s demographic characteristics was used to collect data. All participants completed the questionnaire before the intervention, and they also completed a posttest questionnaire within 2 weeks of finishing the intervention.

The theory-based questionnaire was prepared by reviewing other questionnaires applied in similar studies [15,26,36,37]. Three nutrition and statistical professionals reviewed and revised the questionnaire. The questionnaire was also tested by 15 mothers who had a child aged 1–6 years. Questionnaire items were tested for consistency and comprehensibility. The questionnaire included the following sections; demographic characteristics of the mothers, including age, education (≤high school, undergraduate or ≥graduate), medical background (mother were health professional, such as medical doctors, dietitians or nurses: yes or no), parity (child was firstborn or non-firstborn), household income (≤NT $50,000 or >NT $50,000) and the child’s age. Second, data on the mother’s behaviors and intentions were collected. Behaviors: I used nutrition labels to buy low-sugar foods for my child/children during the past week? (always, usually, sometimes or seldom); Behavioral intentions: I am going to use nutrition labels to buy low-sugar foods for my child/children in the coming week. The third part was about the mothers’ sugar-related knowledge. It included “sugar and health”, “sugar and carbohydrates”, “daily sugar recommendation”, “no-added-sugar claims” and “nutrition labels”. The last part included mother’s attitudes, subjective norms and perceived behavioral control. There were four questions about attitudes (e.g., I believe that using nutrition labels to buy low-sugar foods for my child/children is very important), three question about subjective norms e.g., My family members expect me to use nutrition labels to buy low-sugar foods for my child/children) and four questions about perceived behavioral control (e.g., It is difficult for me to use nutrition labels to buy low-sugar foods for my child/children). A Likert scale was used to score the data collection instruments. In order to avoid neutral feedback, this section used a Likert 6-point scale divided into “very disagree”, “disagree”, “disagree a little”, “consent a little”, “agree” and “very agree”. These were scored 1–6 points, respectively [38].

### 2.5. Data Analysis

A statistical software package (SPSS, Chicago, IL, USA) was utilized. The Kolmogorov-Smirnov test was used to examine the normal distribution of all continuous variables (the test of normality was present at Supplementary Table S1). Nonparametric statistics were used due to most variables did not match normal distribution. Baseline data for demographic characteristics and TPB variables of the two groups were analyzed using a chi-squared and Mann-Whitney U test. A Wilcoxon signed-rank test was used to study changes before and after the educational intervention and a Mann–Whitney U test was used to evaluate the mean of changes and compare the mean of study variables in the two groups. Spearman’s rank correlation was used to examine the correlation among the difference of TPB variables. Statistical significance was set as *p* < 0.05.

## 3. Results

Table 1 shows the demographic characteristics of participants. There was no significant difference in demographic characteristics between the two groups (*p* > 0.05). The mothers’ average ages were 35.3 ± 4.5 years in the online-only group and 34.9 ± 4.5 years in the plus group. The children’s average ages were 2.7 ± 1.6 years in the online-only group and 2.9 ± 1.6 years in the plus group. Most mothers of the two groups had more than one child, had a university/college degree, worked full-time. The average family monthly income was NT $30,000–50,000 and more than 80% of the mothers had no medical background, such as medical doctors, dietitians or nurses.

Table 2 presents the results after the educational intervention, including changes in nutrition knowledge and TPB variables in the two groups and differences between the online-only and plus groups. After the educational intervention, sugar and nutrition label knowledge in both groups significantly improved (*p* < 0.001). The change in the plus group was greater than that in the online-only group (4.3 ± 2.4 vs. 2.0 ± 2.3, *p* < 0.001). No significant difference was observed between the groups regarding the mean scores for behavioral attitudes, perceived behavioral control or subjective norms before the intervention. Mean changes in scores for behavioral attitudes, perceived behavioral control, and subjective norms were significant in the plus group (*p* < 0.05), but the change in behavioral attitudes in the online-only group was not. The mean change in perceived behavioral control in the plus group was greater than that in the online-only group (0.3 ± 0.7 vs. 0.7 ± 0.9, *p* = 0.026). After the intervention, the mean changes in intentions and behavior significantly improved in both groups. The improvement in behavior for the plus group was significantly greater than that of the online-only group (1.8 ± 1.7 vs. 0.8 ± 1.7, *p* = 0.005).

**Figure 3:**
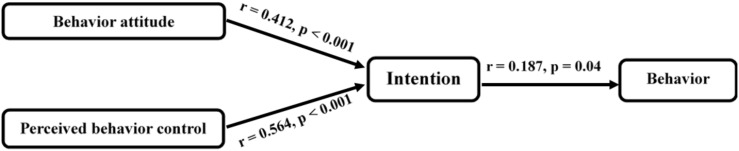
Constructs of the theory of planned behavior in this study (*n* = 122).

Figure 3 presents the correlation among changes in TPB constructs. The figure indicates significant predictive associations with mothers’ intentions to use nutrition labeling to buy low-sugar food and the positive associations between mothers’ intentions and behaviors.

Table 3 presents changes in the mothers’ sugar-related knowledge in both groups after the intervention. The correct rate of all questions had increased in the plus group, but not in the online-only group. Both before and after the intervention, more than 90% of mothers in both groups knew of the association of sugar with health. After the intervention, the change in the correct rate of “No-added-sugar claim” had increased 25% in the online-only group and more than 50% in the plus group. In particular, the correct rate of “comparing sugar contents of foods with ‘no added sugar’ and those without ‘no added sugar’ on the label” had increased 68.7% in the plus group. In the small discussion group session, most mothers expressed that they really cared about the sugar content of foods and they would buy the food with “no added sugar” claim, since they did not realize the difference between “sugar-free” claim and “no added sugar” claim. During the small-group discussion session, they practiced reading several child food packages to understand the difference and found it was important to read the sugar content on nutrition labels.

After the intervention, the correct rates of “sugar and nutrition” exceeded 80% in both groups, and the correct rates of “brown sugar is healthier than white sugar” were still lower than 40% in both groups. The “sugar recommendation” had the lowest correct rates in both groups. After the intervention, changes in the correct rate of “calories from daily sugar intake” was 59.4% in the plus group and only 15.6% in the online-only group. Even after the intervention, correct rates of the other two questions about “sugar recommendations” was still <40% in both groups. During the small-group discussion, most mothers expressed that they knew that the recommended sugar intake differed by age, but it was difficult to memorize different sugar recommended gram for different ages.

**Figure 4:**
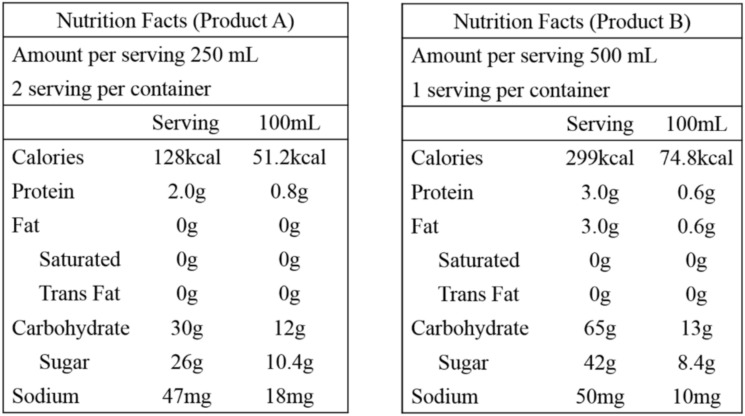
Nutrient labeling information for knowledge part E. nutrition labeling.

After the intervention, the correct rates of “nutrition labeling” were >80% for the online-only group and >75% for the plus group. During the small-group discussion, some mothers expressed confusion about the information presented by nutrition labels. Mothers were confused why some products contained only one serving while other products contained two servings (Figure 4) and they had trouble calculating sugar contents or calories for all products and comparing or choosing products.

## 4. Discussion

### 4.1. Application of the TPB in a Low-Sugar Educational Intervention

This study employed the behavior attitude and perceived behavior control elements of the TPB to design an online educational intervention program. The study results revealed that at one week after participation in the intervention program, both the plus group and online-only group showed increases in intentions and behavioral frequencies toward using nutrition labels to buy food products with lower sugar content for their children. Moreover, changes in behavioral attitudes and perceived behavioral control were positively associated with mothers’ intentions. According to the TPB, behavioral attitudes and perceived behavioral control are crucial factors influencing the behavioral intentions and actual behaviors of people [23]. By applying this theory in the context of this study, it was possible to use an educational intervention to increase the health-related behavioral intentions of participants, which in turn promoted positive behavioral attitudes and ultimately increased actual positive health behaviors. The results of this study supported that educational interventions could influence a person’s behavioral intentions; the stronger the personal behavioral intention is, the greater is the frequency of health behavior execution. Approximately 10% of Taiwanese women aged 25–44 years have at least a master’s-level education [34]. Compared with women’s education level in Taiwan, more participants in this study, particularly in the plus group, had a master’s-level education. Future interventions should be modified to enable more mothers to benefit from the online class.

### 4.2. Advantages of the Online Nutritional Education Intervention

In considering both the pervasiveness of Internet services and mothers with young children may have difficulties attending face-to-face learning sessions, this study used online videos to deliver the intervention program; some mothers who were interested could participate in the small-group discussion sessions. The online learning program seemed to be a suitable educational intervention tool for the mothers who participated in this study were highly educated (90% had greater than a college educational level). A study conducted in the US revealed that online teaching media (e.g., emails and websites) are low-cost intervention tools for nutrition education, and more important, these online teaching media can enhance the willingness of learners to participate [39]. The completion rate of this study was 62.1% for the online-only group and 80.0% for the plus group. This finding indicated that although online videos could increase the mothers’ convenience to participate in the intervention program, some mothers still had insufficient time available to finish watching two 15-minute online videos. As for the plus group, the participants may have had stronger intentions and motivation to learn and were therefore willing to overcome obstacles to complete the intervention program. The online videos used in the present study should be modified to increase mothers’ participation rates; for instance, the videos should be split into shorter, more-manageable videos, and also the content could be modified according to results of the group discussions in the present study.

This study revealed that compared to the online-only group, the plus group consciously enhanced their perceived behavioral control and frequency of using nutrition labels to buy low-sugar food products after participating in the intervention program. This indicated that the small-group discussions can improve the ability of mothers to read and use nutrition labels. A study conducted in the UK revealed that group interventions are useful for teaching mothers about child feeding techniques and helping them achieve desired dietary targets for their children [40]. In another study analyzing parents as nutritional education participants, group discussions had a positive role in remedying parents’ difficulties with feeding their children and improving their feeding techniques [41]. Although the small-group discussions are a useful intervention tool, it was difficult to identify a group meeting time that suited three or more participants in this study, because more than half of the participants were working mothers. Therefore, eight participants (20%) of the plus group could not even complete one required face-to-face discussion session. Because an increasing number of web conferencing software programs, such as Skype business, are becoming popular, future studies may use this kind of software to conduct the small-group discussions and increase the participation rate of mothers.

### 4.3. Improvement of Mothers’ Knowledge of Nutrition Labels

Results of the current study revealed that after the intervention, the knowledge, behavioral attitudes, perceived behavioral control, intentions and behaviors of the plus group participants had significantly improved. Specifically, their knowledge, perceived behavioral control, and behaviors significantly improved compared to those of the online-only group. Regarding the plus group, in addition to watching the online educational videos, they participated in a small-group discussion to clarify doubts regarding the videos and engage in nutritional-label-use practices. Both their behavioral attitudes and perceived behavioral control in terms of using nutrition labels to shop for low-sugar foods improved after the intervention. A previous study found that nutrition knowledge can improve the ability of participants to shop for healthy food items [42], while another study demonstrated that knowledge influences purchasing behaviors through its influence on attitudes [43]. Berg et al. [44] asserted that behavioral attitudes and perceived behavioral control are crucial factors that influence personal food choice intentions. A study in Iran that employed the TPB to increase participants’ fruit and vegetable intake revealed that compared to pre-intervention levels, participants’ post-intervention behavioral attitudes, perceived behavioral control, and behavioral intentions significantly improved [45]. However, the researchers asserted that even if a person had positive behavior attitudes toward such food items, if he or she did not possess the ability to implement autonomous control, then they would still be unable to modify their behavior [45]. Results of a study about fruit and vegetable intake also confirmed that perceived behavioral control is a fundamental factor in predicting food choice intentions and behaviors [46]. Results of the present study revealed that after the educational intervention was administered, mothers in both groups had a significantly improved ability to consciously shop for low-sugar foods using nutrition labels. This indicates that enhancing perceived behavioral control is an important factor in increasing participants’ positive nutrition-label-use behaviors.

Although the present study did not incorporate subjective norms into the design of the educational program, the subjective norm scores of both groups significantly increased from the pre- to post-intervention stages. It is possible that during the study period, participants may have engaged in additional discussions related to sugar, health, and/or nutrition labels with their family and friends, which subsequently led to an increase in support from friends and family with regard to controlling the sugar intake of their children. This would ultimately have led to an increase in subjective norm scores. To further increase the support of family and friends towards participants, in future studies, intervention sessions could be designed in which the participants could engage in discussions with their friends and family.

### 4.4. Understanding No-Added-Sugar Claims and Changing Behavioral Attitudes

In terms of behavioral attitudes, this study demonstrated that before the educational intervention was implemented, fewer than 50% of the online-only group participants and fewer than 40% of the plus group had correct perceptions about the “no-added-sugar” claim, whereas more than 50% of the total participants believed that food items with the “no-added-sugar” label contained less sugar than food items without it. During the group discussions, several participants reported that they would buy the “no-added-sugar” food items for their children, but they did not know considered the actual sugar content. A survey conducted in Taiwan reported that more than half of mothers believed that infant cereals with “no-added-sugar” claims contained less sugar than products without such claims [15]. After the educational intervention was implemented, the mean percentage of correct responses improved by 25.0% for the online-only group and 56.3% for the plus group. A study conducted in the UK revealed that approximately 40% of responding parents took the initiative to search for food items with the “no-added-sugar” label when shopping for food for their children [47]. Furthermore, a snack and beverage marketing survey conducted in Taiwan also revealed that more than 90% of children’s popular snacks and beverages with “no-added-sugar” or “no-artificial-sweeteners” claims actually had high sugar contents [13]. If parents do not possess sufficient knowledge about sugar claims, they may be easily misled by the “no-added-sugar” claims and end up choosing food items that actually have high sugar contents. In the future, stricter sugar claims regulations should be implemented to prevent the public from being misled. Additionally, the present study revealed that mothers’ incorrect perceptions could be effectively modified using online videos and/or group discussions. Furthermore, group discussions can enhance mothers’ positive attitudes toward paying attention to nutrition labels. To promote the effects of online videos, future interventions could include explanations (with relevant examples) about “no-added-sugar” claims and sugar contents of common foods.

### 4.5. Awareness of Sugar Recommendations

In this study, although 90% of mothers in this study had a college education, after the intervention, about 40% of the plus group and 80% of the online-only group did not know the WHO sugar recommendations. These findings are consistent with the previous study that even highly educated people are rarely aware of sugar recommendations [48]. An online survey in Northern Ireland found that even about 90% of participants were college level, there were still 65% of them were unaware of the WHO guidelines for sugar intake, only 4% of respondents correctly classified sugar and artificial sweeteners [48]. Another online survey among Canadian young people also found that only 4.8% of participants correctly identified Canadian recommendations for sugar intake [49]. The present study also found that during the small-group discussion, mothers mentioned the difficulty of remembering WHO recommendations or were confused about the different acceptable daily sugar intake for different age groups. Understanding sugar recommendations and using nutrition labels to choose foods are important to help people control their sugar intake. Therefore, more nutrition education or campaign are needed to raise people’s awareness of the sugar recommendations and control their sugar intake.

### 4.6. Difficulties with Nutrition Labels

The study found that after the intervention, about 15% of both groups still had difficulty interpreting nutrition labels. During the group discussion, the mothers expressed difficulties in using nutrition labels to calculate sugar contents and selecting foods with low-sugar contents. Mothers were confused why some products contain only one serving, but other products contain more than two servings thus they had trouble using nutrition labels to compare or determine sugar contents of products. An experiment about the efficacy of nutrition label formats found that the percent daily value information could help consumers correctly identify the relative amount of total sugar, and the added sugar information could help people correctly identify the relative amount of added sugar in a food [49]. A previous web-based label-reading training intervention also found that web-based practice led to improvements in nutrition label-reading skills; however, consumers may not bother using nutrition labels if reading them is too difficult or time-consuming [21]. Previous studies showed that graphic symbols, such as a traffic light, were easily understood by people with different education or income levels [50,51,52]. In Taiwan, the percent daily value of sugar or graphic symbols are not available. Further studies are needed to develop the simple label to help parents to choose the low sugar food.

### 4.7. Limitations and Further Developments

First, although the online video course made it convenient for mothers with young children to participate, it is not possible to confirm whether all the mothers watched and understood all of the online videos. Second, the importance of knowledge on sugar and intention to use food labels for mothers’ food purchasing decisions remains unclear. Third, because the groups were assigned by participant preference, the motivation of those in the plus group may have been higher than that in the online-only group. This may have been a factor causing differing results between the two groups.

The results suggest that an online intervention increases mothers’ intentions to use nutrition labeling to buy low-sugar foods for their children. Online video s should be improved using the feedback from small-group discussions; therefore, more mothers can benefit from online classes. Furthermore, more research with a larger sample size is warranted to evaluate the effects of the intervention on long-term changes in mothers’ real shopping behaviors. Purchases over one week after the intervention may be an inadequate reflection of change. Therefore, an examination of purchases over time, such as one or two months later, is required.

## 5. Conclusions

This study demonstrated that after the online sugar educational intervention, mothers in both the online-only and plus groups exhibited increased intentions and behavioral frequencies of using nutrition labels when selecting low-sugar food products for their children. In addition, after the intervention, approximately 40.0% of the plus group and 80% of the online-only group still did not know the WHO sugar recommendations. Awareness of the WHO sugar recommendations and using nutrition labels to select foods are instrumental in helping people control their calorie and sugar intake. Therefore, more efficient and convenient nutrition education is required to increase public awareness of sugar recommendations and help people control their calorie and sugar intake.

## Figures and Tables

**Table 1:** Demographic characteristics of participants by the intervention condition ^1^.

Characteristics	Total (*n* = 122)	Online Only Group (*n* = 90)	Plus Group ^2^ (*n* = 32)	*p* Value
Mother’s Age (years)	35.3 ± 4.5	35.3 ± 4.5	34.9 ± 4.5	0.456
Child’s Age (years)	2.8 ± 1.6	2.7 ± 1.6	2.9 ± 1.6	0.573
Number of Children				0.270
One	52 (42.6)	41 (45.6)	11 (34.4)	
More than One	70 (57.4)	49 (54.4)	21 (65.6)	
Education				0.241
Senior High School	13 (10.7)	9 (10.0)	4 (12.5)	
University/College	84 (68.8)	65 (72.2)	19 (59.4)	
≥Master’s	25 (20.5)	16 (17.8)	9 (28.1)	
Working status				0.605
Housewife	47 (38.5)	36 (40.0)	11 (34.4)	
Full-time	61 (50.0)	44 (48.9)	17 (53.1)	
Part-time	14 (11.5)	10 (11.1)	4 (12.5)	
Medical Background				0.398
No	107 (87.7)	79 (87.8)	28 (87.5)	
Yes	15 (12.3)	11 (12.2)	4 (12.5)	
Family Income, NT$/month ^3^				0.718
<30,000	16 (13.1)	11 (12.2)	5 (15.6)	
30,001–50,000	33 (27.0)	25 (27.8)	8 (25.0)	
50,001–70,000	28 (23.0)	22 (24.4)	6 (18.8)	
70,001–100,000	26 (21.3)	20 (22.2)	6 (18.8)	
≥100,001	19 (15.6)	12 (13.3)	7 (21.9)	

^1^ Data are presented as the number (percentage) or mean ± standard deviation; ^2^ participants of the plus group finished online videos and a group discussion; ^3^ the average exchange rate in 2018 was US1.00 ≈ New Taiwan (NT) $30.

**Table 2:** Changes in knowledge and theory of planned behavior (TPB) before and after the intervention in both groups. ^1.^

Variable	Online-Only Group	Plus Group ^2^	*p* Value ^3^
	Mean ± SD	Mean ± SD	
Knowledge of Sugar and Labels (0–16 score)			
Before	9.4 ± 2.0	8.3 ± 2.2	0.039 *
After	11.4 ± 2.1	12.6 ± 1.6	0.006 *
Difference	2.0 ± 2.3	4.3 ± 2.4	<0.001 **
*p* value ^4^	<0.001 **	<0.001 **	
Behavioral Attitudes (1–6 score)			
Before	5.0 ± 0.8	4.9 ± 0.7	0.351
After	5.1 ± 0.7	5.3 ± 0.7	0.294
Difference	0.2± 0.7	0.3 ± 0.9	0.071
*p* value ^4^	0.166	0.030 *	
Perceived Behavioral Control (1–6 score)			
Before	4.6 ± 0.8	4.6 ± 0.7	0.713
After	5.0 ± 0.7	5.3 ± 0.6	0.056
Difference	0.3 ± 0.7	0.7 ± 0.9	0.026 *
*p* value ^4^	<0.001 **	<0.001 **	
Subjective Norms (1–6 score)			
Before	4.5 ± 1.0	4.4 ± 0.9	0.375
After	4.8 ± 1.0	4.7 ± 0.8	0.197
Difference	0.3 ± 0.9	0.3 ± 0.8	0.965
*p* value ^4^	0.001 *	0.035 *	
Behavioral Intentions (1–6 score)			
Before	5.1± 1.0	4.9 ± 0.9	0.122
After	5.4 ± 0.7	5.4 ± 0.6	0.636
Difference	0.3 ± 0.8	0.5 ± 0.8	0.054
*p* value ^4^	0.001 *	0.002 *	
Behaviors (1–5 score)			
Before	2.8 ± 1.7	2.1 ± 1.6	0.040 *
After	3.5 ± 1.5	3.9 ± 1.3	0.356
Difference	0.8 ± 1.7	1.8 ± 1.7	0.005 *
*p* value ^4^	<0.001 **	<0.001 **	

^1^ Data are presented as mean and standard deviation (SD); ^2^ participants of the plus group finished online videos and a group discussion; ^3^ difference between the online-only group and plus group; ^4^ difference between the before and after scores; * *p* < 0.05; ** *p* < 0.001 by Mann-Whitney U test and Wilcoxon signed-rank test.

**Table 3:** Correct knowledge rates about sugar and nutrition labels in both groups ^1^.

Section Question	Answer	Online-Only Group (*n* = 90)			Plus Group (*n* = 32)		
		Before	After	Change (%)	Before	After	Change (%)
A. Sugar and Health Average Rate		96.3	97.1	0.8	96.9	99.0	2.1
High sugar intake increases the risk of obesity.	T	89 (98.9)	88 (97.8)	1.0	31 (96.9)	32 (100.0)	1.0
High sugar intake increases the risk of tooth decay.	T	88 (97.8)	87 (96.7)	−1.1	32 (100.0)	32 (100.0)	0.0
High sugar intake increases the preference for sweets.	T	83 (92.2)	87 (96.7)	4.5	30 (93.8)	31 (96.9)	3.1
B. No-added-sugar Claims		48.9	73.9	25.0	29.7	86.0	56.3
“No added sugar” signifies “sugar free”.	F	61 (67.8)	77 (85.6)	17.8	16 (50.0)	30 (93.8)	43.8
The sugar content of food with “No added sugar” is lower than food without “no added sugar”.	F	27 (30.0)	56 (62.2)	32.2	3 (9.4)	25 (78.1)	68.7
C. Sugar and nutrition		55.9	77.0	21.1	46.9	82.0	35.1
Whole-grain foods and sugar are carbohydrates.	T	63 (70.0)	78 (86.7)	16.7	20 (62.5)	29 (90.6)	28.1
Honey is one type of sugar.	T	61 (67.8)	78 (86.7)	18.9	23 (71.9)	32 (100.0)	28.1
Sugar contains 4 calories per gram.	T	51 (56.7)	86 (95.6)	38.9	15 (46.9)	32 (100.0)	53.1
Brown sugar is healthier than white sugar.	F	26 (28.9)	35 (38.9)	10.0	2 (6.3)	12 (37.5)	31.2
D. Sugar recommendations		3.3	13.7	10.4	2.1	39.6	37.5
Calories from daily sugar intake should be greater than 20%of total calories.	F	3 (3.3)	17 (18.9)	15.6	1 (3.1)	20 (62.5)	59.4
The sugar content of one Yakult ^2^ is higher than the daily sugar recommendation for 1–3-year-old children.	F	1 (1.1)	3 (3.3)	2.2	0 (0.0)	6 (18.8)	18.8
Fifty grams of sugar of intake/per day is acceptable for 4–6-year-old children.	F	5 (5.6)	22 (24.4)	18.8	1 (3.1)	12 (37.5)	34.4
E. Nutrition labels		79.6	87.5	7.9	71.9	86.0	14.1
Product A contains 26 g of sugar.	F	65 (72.2)	72 (80.0)	7.8	26 (81.3)	28 (87.5)	6.2
Product B contains 65 g of carbohydrates.	T	74 (82.8)	90 (100.0)	17.2	23 (71.9)	31 (96.9)	25.0
Product A contains fewer calories than product B.	T	75 (83.3)	77 (85.6)	2.3	20 (62.5)	24 (75.0)	12.5
Product B contains a lower sugar content than product A.	T	72 (80.0)	76 (84.4)	4.4	23 (71.9)	27 (84.4)	12.5

^1^ Data are presented as the number (percentage); ^2^ a popular yogurt drink; T: True; F: False.

## Supplementary Materials

The following are available online at https://www.mdpi.com/2072-6643/12/6/1859/s1, Table S1: Test of normality for knowledge and constructs of TPB.
