# Supplementary material for: The Effect of an Online Sugar Fact Intervention: Change of Mothers with Young Children
Source: Nutrients. 2020 Jun 22;12(6):1859. doi: 10.3390/nu12061859 (PMC7353269; doi:10.3390/nu12061859)
Supplement: Supplementary file 1 [file nutrients-12-01859-s001.pdf]

Supplement table S 1. Test of normality for knowledge and constructs of TPB.

	Online-only group		Plus group	
**Variable**	**Statistics**	**Sig.**	**Statistics**	**Sig.**
Knowledge of sugar and labels				
Before	0.123	0.002	0.153	0.055
After	0.119	0.003	0.140	0.112
Behavioral attitudes				
Before	0.159	<0.001	0.171	0.018
After	0.181	<0.001	0.194	0.004
Perceived behavioral control				
Before	0.094	0.047	0.163	0.031
After	0.110	0.009	0.180	0.010
Subjective norms				
Before	0.140	<0.001	0.168	0.023
After	0.200	<0.001	0.137	0.133
Behavioral intentions				
Before	0.264	<0.001	0.211	<0.001
After	0.345	<0.001	0.328	<0.001
Behaviors				
Before	0.268	<0.001	0.405	<0.001
After	0.288	<0.001	0.342	<0.001
